# Astrocyte senescence may drive alterations in *GFAPα*, *CDKN2A p14*^*ARF*^, and *TAU3* transcript expression and contribute to cognitive decline

**DOI:** 10.1007/s11357-019-00100-3

**Published:** 2019-10-25

**Authors:** Jed J. Lye, Eva Latorre, Ben P. Lee, Stefania Bandinelli, Janet E. Holley, Nicholas J. Gutowski, Luigi Ferrucci, Lorna W. Harries

**Affiliations:** 1 Institute of Biomedical and Clinical Sciences, University of Exeter Medical School, Exeter, Devon EX2 5DW UK; 2 Geriatric Unit, USL Toscana Centro, Florence, Italy; 3 University of Exeter Medical School & Neurology Department, Royal Devon & Exeter Hospital, Exeter, EX2 5DW UK; 4 National Institute on Aging, Clinical Research Branch, Harbor Hospital, Baltimore, MD 21225 USA

**Keywords:** Neurodegenerative disease, Alternative splicing, Senescence, Gene expression

## Abstract

The accumulation of senescent cells in tissues is causally linked to the development of several age-related diseases; the removal of senescent glial cells in animal models prevents Tau accumulation and cognitive decline. Senescent cells can arise through several distinct mechanisms; one such mechanism is dysregulation of alternative splicing. In this study, we characterised the senescent cell phenotype in primary human astrocytes in terms of SA-β-Gal staining and SASP secretion, and then assessed splicing factor expression and candidate gene splicing patterns. Finally, we assessed associations between expression of dysregulated isoforms and premature cognitive decline in 197 samples from the InCHIANTI study of ageing, where expression was present in both blood and brain. We demonstrate here that senescent astrocytes secrete a modified SASP characterised by increased IL8, MMP3, MMP10, and TIMP2 but decreased IL10 levels. We identified significant changes in splicing factor expression for 10/20 splicing factors tested in senescent astrocytes compared with early passage cells, as well as dysregulation of isoform levels for 8/13 brain or senescence genes tested. Finally, associations were identified between peripheral blood *GFAPα*, *TAU3*, and *CDKN2A* (*P14*^*ARF*^) isoform levels and mild or severe cognitive decline over a 3–7-year period. Our data are suggestive that some of the features of cognitive decline may arise from dysregulated splicing of important genes in senescent brain support cells, and that defects in alternative splicing or splicing regulator expression deserve exploration as points of therapeutic intervention in the future.

## Introduction

Age is a major risk factor for cognitive decline and neurodegenerative disease, as it is for most common, chronic disorders (Niccoli and Partridge, 2012). The accumulation of senescent cells in tissues and organs has emerged as a major driver of the ageing process and age-related disease in mammals; selective removal of such cells in genetically engineered animals has been demonstrated to reverse or delay aspects of ageing (Baker et al., 2016, Baker et al., 2011). Senescent cells are alive and metabolically active, but have altered function compared with their non-senescent counterparts. A major feature of senescence is secretion of the senescence-associated secretory phenotype (SASP) which can act in a paracrine manner on neighbouring cells to drive organismal ageing (Freund et al., 2010). Although terminally differentiated cells such as neurones may not undergo replicative senescence in the accepted sense, other important support cells in the brain such as astrocytes that do have proliferative capacity are subject to this phenomenon. Astrocytes have a pivotal role in maintenance of trophic factors, neurotransmitters, and the support and strengthening of neuronal connections (Sidoryk-Wegrzynowicz et al., 2011). Previous evidence has suggested that the accumulation of senescent astrocytes may drive neurodegenerative disorders (Di Malta et al., 2012) and recent work shows that clearance of senescent glial cells may prevent the accumulation of Tau aggregates and improve cognitive function in mouse models (Bussian et al., 2018, Baker and Petersen, 2018). Pharmacological impairment of astrocytic function recapitulates cognitive deficits that are observed in old age (Tarantini et al., 2017). It is also noteworthy that irradiation-induced accumulation of senescent cells is associated with cerebromicrovascular dysfunction in animal models (Ungvari et al., 2017).

Cells enter a senescent state for a number of reasons, one of which is repeated exposure to internal and external stressors (de Magalhaes and Passos, 2018). Exposure to cellular stress elicits an adaptive and plastic transcriptional response, which is partly orchestrated by alternative splicing (Mastrangelo et al., 2012). Alternative splicing is mediated by the combinatorial binding of a series of splicing activators and inhibitors to splicing enhancer and silencer sequences around the splice sites to determine whether or not each splice site is used (Fu and Ares, 2014, Smith and Valcarcel, 2000). Supporting the notion of a pivotal role for dysregulated alternative splicing in senescence, transcripts associated with chronological age in humans are enriched in gene ontology pathways involved in splice site choice (Harries et al., 2011) and splicing factor expression is also associated with cellular senescence in in vitro models (Holly et al., 2013), with human ageing phenotypes (Lee et al., 2019) and with lifespan in long-lived mice (Lee et al., 2016). Perhaps most persuasively, restoration of splicing factor levels is associated with rescue of cellular senescence in multiple human primary human cell types (Latorre et al., 2017, Latorre et al., 2018a, Latorre et al., 2018c). Changes to the regulation of splicing have previously been reported in Alzheimer’s Disease (Wong, 2013) and global dysregulation of splicing is a characteristic of several neurodegenerative conditions such as Huntington’s disease (Lin et al., 2016), frontotemporal lobar dementia (Gao et al., 2017), and Parkinson’s Disease (Soreq et al., 2012). Specially, alterations to the splicing pattern of the *MAPT* gene which encodes Tau protein are known to contribute to neurofibrillary tangles (Goedert and Jakes, 1990).

We hypothesised that senescent astrocytes would display differential expression of splicing regulatory factors and altered patterns of alternative splicing in vitro, and that some of these isoform changes may be detectable in peripheral blood and show statistical associations with cognitive phenotypes in human populations. We first characterised the astrocyte SASP in terms of cytokine and MMP production, and then determined the splicing factor repertoire and patterns of alternative splicing for a panel of brain or senescence candidate genes in senescent human primary astrocytes. Where dysregulation of splicing patterns was demonstrated and expression was conserved in blood, we then assessed associations between peripheral blood isoform levels and measures of cognitive dysfunction from 197 individuals from the InCHIANTI study of ageing, a longitudinal and cross-sectional population study of individuals from the Tuscany region of Italy (Ferrucci et al., 2000). We identified that senescent astrocytes display a modified SASP, consisting of elevated IL8, MMP, MMP10, and TIMP2 levels, but decreased IL10. Of the splicing regulatory factors tested, 50% demonstrated dysregulated expression in senescent astrocytes; this was accompanied by altered splicing of 7/13 of candidate genes tested. Furthermore, when we assessed the relationship between peripheral blood expression of isoforms dysregulated in astrocytes and cognitive decline as measured my Mini Mental State Exam (MMSE), *GFAPα* and *TAU3* transcript levels were positively correlated with cognitive decline, whereas GFAPΑ transcript levels were negatively associated with cognitive decline over a 3–7-year period in participants from the InCHIANTI study of ageing. Our data are in agreement with the hypothesis that senescent astrocytes display differential expression of splicing regulatory factors and altered patterns of alternative splicing, and that some of these isoform changes may reflect those in peripheral blood. Such changes may show statistical associations with cognitive phenotypes in human populations.

## Experimental protocols

### Culture of early and senescent astrocytes

These studies used cultures of early passage and late passage human primary astrocytes (HPA) previously isolated from a block of sub-ventricular deep white matter tissue in a 17-year-old male donor immediately post-mortem with consent from next-of-kin. Ethical approval was granted by the North and East Devon Research Ethics Committee. Astrocytes were isolated from tissue blocks as previously described (Holley et al., 2005). Cells were maintained in humidified incubators with 95% O_2_/5% CO_2_ in HPA stock media (Celprogen Inc., Torrance, CA, USA). For the production of senescent cultures, cells were counted and equal numbers of cells seeded (4 × 10^3^ cell/cm^2^) at each passage in continuous culture until the growth of the culture slowed to less than 0.5 population doublings (PD)/week. Astrocyte cultures underwent continuous culture until the onset of replicative senescence and growth arrest in 3 biological replicates. Early passage astrocytes at PD = 24 and late passage astrocytes at PD = 84 were used.

### Quantification of senescent cell load

Cell senescence was assessed in 3 biological replicates by the biochemical senescence marker senescence-associated β-galactosidase (SA-β-Gal) using a commercial kit (Sigma Aldrich, UK) according to the manufacturer’s instructions, with a minimum of 100 cells assessed per replicate. Senescence was also quantified by assessing the expression of the *CDKN2A* gene (a known molecular marker of cell senescence) and by changes in cell morphology typical of senescence as in our previous work (Latorre et al., 2017, Holly et al., 2013). Total RNA (100 ng) was reverse transcribed in 20 μl reactions using EvoScript reverse transcriptase (Roche Life Sciences, Burgess Hill, UK). Total *CDKN2A* expression was measured by qRT-PCR relative to 3 empirically determined endogenous control genes (*GUSB*, *PPIA*, and *GADPH*) on the QuantStudio 12K Flex platform (Applied Biosystems, Foster City, USA). PCR reactions contained 2.5 μl TaqMan Universal Mastermix (no AMPerase) (Applied Biosystems, Foster City, USA), 900 nM of each primer, 250 nM probe, and 0.5 μl cDNA in a total volume of 5 μl. Cycling conditions were a single cycle of 95 °C for 10 min followed by 40 cycles of 95 °C for 15 s and 60 °C for 1 min.

### Characterisation of the senescence-associated secretory phenotype in early and late passage cells

Cells were seeded in two biological replicates of 10 × 10^4^ cells in a 25-cm^2^ flasks and cultured until 80% confluence. Cell supernatants were then harvested and stored at − 80 °C. The senescence-associated secretory phenotype was then quantified in the culture media following 48 h incubation using the ABCAM Human Cytokine Antibody Array (ab133997; Abcam, Cambridge, UK) and the ABCAM Human MMP Antibody Array (ab134004; Abcam, Cambridge, UK). SASP components measured were as follows: IL-1B, IL-2, Il-6, IL-8, IL-10, TNF-α, IFN-γ, GM-CSF, Angiogenin, ENA78, GRO-α, MMP3, MMP10, and TIMP2. Results were normalised using positive and negative controls as per the kit instructions using the LI-COR Odyssey® CLx imaging system (Lincoln, NE, USA). An unpaired two tailed *t* test was used to assess statistical significance in secreted matrix metalloproteinases and inflammatory cytokine secretion using Minitab 18 software package (Minitab, Centre County, USA).

### Quantification of splicing factor expression

Splicing factors have previously been demonstrated to be associated with cellular senescence with evidence suggesting they may be drivers of this process in some tissues (Latorre et al., 2017, Latorre et al., 2018a, Latorre et al., 2018c). We measured the expression levels of an a priori panel of 20 splicing factors previously associated with age, lifespan, and cellular senescence in different tissue types in our previous work (Harries et al., 2011, Holly et al., 2013, Latorre et al., 2018b). This panel included the splicing inhibitors *HNRNPA0*, *HNRNPA1*, *HNRNPA2B1*, *HNRNPD*, *HNRNPH3*, *HNRNPK*, *HNRNPM*, *HNRNPUL2*, the splicing activators *AKAP17A*, *PNISR*, *SRSF1*, *SRSF2*, *SRSF3*, *SRSF6*, *TRA2B*, *SRSF7* and the *SF3B1*, *IMP3*, *LSM14A*, and *LSM2* components of the core spliceosome*.* Splicing factor expression was measured in 3 biological and 2 technical replicates by qRT-PCR using custom TaqMan Low Density Arrays (TLDA) on the Quantstudio 12K Flex platform as previously described (Holly et al., 2013). Transcript levels were expressed relative to the geometric mean of the *GUSB* and *PPIA* endogenous control genes and normalised to their expression in RNA from early passage cells. We then performed tests for equality of variance and *t* test using IBM SPSS Statistics 25.

### Quantification of candidate gene expression in early passage and senescent astrocytes

A panel of candidate genes were selected for analysis on the basis of biological relevance (known links with brain function, neurodegenerative disease, or senescence) and where available evidence from the literature that alternatively expressed isoforms may have differential function to allow interpretation of changes. The identity of genes tested and the rationale for their inclusion are given in Table 1. TaqMan Assays specific to particular isoforms were designed to unique regions of the transcripts in question (assay sequences are available upon request). Assays were validated by standard curve analysis using 7 serial 1:2 dilutions of cDNA derived from the whole brain lysate. Reverse transcription and qRT-PCR conditions are described above. Experiments were carried out in 3 biological and 3 technical replicates. Transcript levels were expressed relative to *GUSB* and *PPIA* endogenous control genes and normalised to their expression in RNA from early passage cells. We then performed tests for equality of variance and *t* test using IBM SPSS Statistics 25.

**Table 1:** Transcript isoforms identified for expression analysis

Gene	Transcript accession	Isoform/transcript function
ATM	NM_000051.3	DNA damage repair
AQP4		
*AQP4M1*	NM_001650.6	Pore-forming integral membrane protein
*AQP4M23*	NM_004028.4	Pore-forming integral membrane protein
SLC1A2		
*EAAT2A*	NM_004171.4	Excitatory amino acid transporter
*EAAT2B*	NM_001252652.1	Excitatory amino acid transporter
GFAP		
*GFAPΑ*	NM_002055	Astrocyte intermediate filament protein
*GFAP*(*B*)	NM_001131019.1	Astrocyte intermediate filament protein
KL		
*KLOTHO mKl*	NM_004795	Membrane bound isoform, coreceptor for FGF23
*KLOTHO msKl*	NM_004795.3	Secreted isoform, endocrine factor which improves cognitive performance in ageing
CDKN2A		
*p14*^*ARF*^	NM_058195	p53 pathway to cell cycle cessation
*p16*^*INK4A*^	NM_001195132	RB1 pathway to cell cycle cessation (Takahashi et al., 2007)
CDKN1A		
*p21a*	NM_078467	Inhibits proliferation
*p21b*	NM_000389	Promotes proliferation
TP53	NM_001126118, NM_000546, NM_001126112, NM_001276696, NM_001126113, NM_001276699, NM_001276698, NM_001276697	Cell cycle regulation
PSEN2	NM_012486.2	Processing of β-amyloid
MAPT		
*TAU3*	NM_001203251.1 NM_001203252.1 NM_016841.4	Microtubule protein involved in neurofibrillary tangles
*TAU4*	NM_001123066.3 NM_001123067.3 NM_005910.5 NM_016834.4 NM_016835.4	Microtubule protein involved in neurofibrillary tangles

The table gives the identity of the isoforms selected for analysis, the relevant NM accession numbers, and a brief description of their function

### Assessment of candidate gene expression levels with cognitive decline in a longitudinal human population

The InCHIANTI study of ageing is a population study of ageing with detailed assessment of health and lifestyle parameters at baseline, and again at 4 subsequent follow-ups (FU2; 2004–2006, FU3; 2007–2009, and FU4; 2012–2014) (Ferrucci et al., 2000). We selected 197 participants for study. Inclusion criteria were age at FU3 > 64 years with an MMSE score > 18 to avoid those already on a declining cognitive trajectory and availability of an FU3 RNA sample with clinical information available at both FU3 and FU4. Participants were categorised into ‘mild’ or ‘severe’ groups depending their change in the MMSE score between FU3 and FU4; individuals declining between 2 and 8 points were defined as ‘mild’, whereas those declining between 9 and 22 points were categorised as ‘severe’. These thresholds were chosen on the basis of previously defined criteria, where a ‘severe’ decline was categorised as a drop in MMSE score > 3 points per annum (Clark et al., 1999, McCarten et al., 2004, Hensel et al., 2007). Ethical approval was granted by the Instituto Nazionale Riposo e Cura Anziani institutional review board in Italy. Methods were carried out in accordance with the relevant guidelines and regulations. Informed consent was obtained from all participants.

Blood was collected into PAXgene tubes (BD Biosciences) at FU3 and extracted using the PAXgene blood RNA kit (Qiagen, Paisley, UK). One hundred nanograms RNA was then reverse transcribed using the EvoScript Universal cDNA Master kit (Roche Life Sciences, Burgess Hill, UK) according to the manufacturer’s instructions except for a change to the extension phase of the reaction: a step of 30 min at 65 °C was used instead of 15 min at 65 °C. Expression levels for transcripts showing senescence-related dysregulation in levels in astrocytes were quantified in peripheral blood as described above. Samples were assessed in 3 technical replicates. Data were log transformed and multivariate linear regression models were performed using STATA© SE 15 to investigate the association between transcript expression and cognitive decline as measured by change (Δ) in MMSE score. Models were adjusted for age, gender, smoking (lifetime pack-years), study site, education level, and white blood cell subtype counts (% neutrophils, monocytes, basophils, eosinophils).

## Results

### Characterisation of senescent astrocytes

**Fig. 1:**
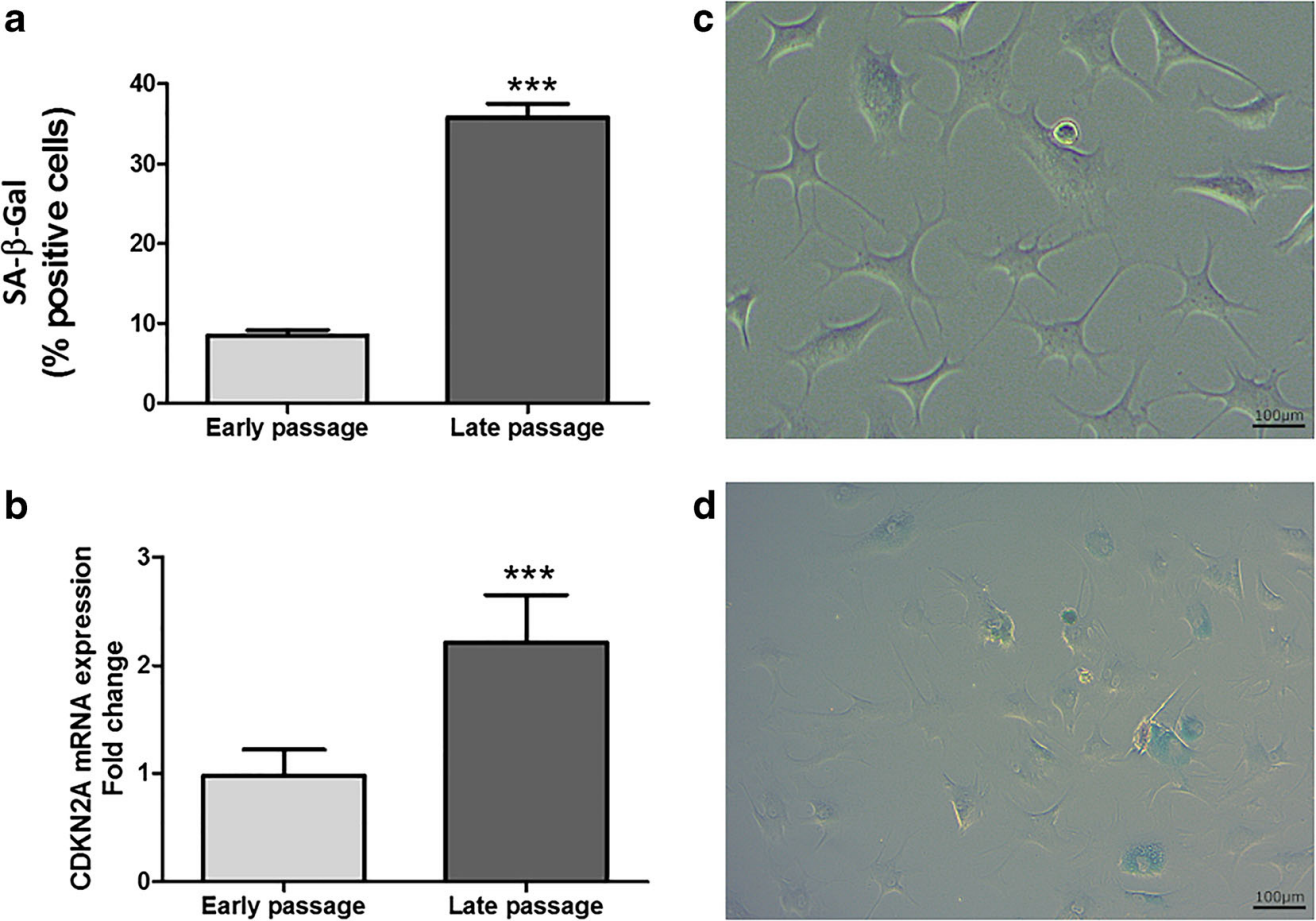
Senescent cell quantification in early passage and late passage human primary astrocytes. a This graph gives the change in senescent cell load as determined by senescence-associated β-galactosidase (SA-β-Gal) staining between early passage and late passage human primary astrocytes. The percentage of cells staining positive for SA-β-Gal is given on the *Y* axis and the identity of the cell culture on the *X* axis. **b** Change in senescent cell load as determined by *CDKN2A* expression between early passage and late passage human primary astrocytes. Levels of *CDKN2A* transcripts relative to the endogenous control genes (*GUSB*, *PPIA*, and *GADPH*) and normalised to the expression levels in early passage cells are given on the *Y* axis and the identity of the cell culture on the *X* axis. Early passage cells are population doubling (PD) 24; late passage cells are at PD = 84. Results are from 3 biological replicates. *p < 0.01, **p < 0.01, ***p < 0.001. Error bars refer to standard deviation of measurement. **c** Senescence-associated β-galactosidase staining in early passage astrocytes (PD 24). **d** Senescence-associated β-galactosidase staining in late passage astrocytes (PD 84)

Human primary astrocyte cultures were considered senescent at PD84 at which point they were considered senescent. This was verified by molecular and biochemical characterisation of the growth kinetics of the cultures; senescence-associated β-galactosidase (SA-β-gal) staining demonstrated a significant increase in the number of senescent cells from 8% in early passage cells to 36% in late passage cells (Fig. 1a) which was mirrored by a concurrent increase in the expression of the *CDKN2A* gene (Fig. 1b). SASP factors in conditioned media derived from senescent cells demonstrated altered levels for several key SASP proteins; we observed elevated IL-8, GM-CSF, Angiogenin, ENA78, GRO-α, MMP-3, MMP-10, and TIMP2 levels, but reduced IL-10 levels (Table 2).

**Table 2:** Evaluation of astrocyte SASP. Level of secreted cytokines and matrix metalloproteinase proteins in senescent and non-senescent human primary astrocytes is given below

Cytokine	Early passage (SE)	Late passage (SE)	*p* value
IL-1B	274.233 (24.37)	286.058 (36.82)	0.798
IL2	74.659 (23.99)	111.541 (18.97)	0.273
IL-6	78.752 (11.81)	73.490 (9.49)	0.740
*IL-8*	**409.541 (55.96)**	**1097.730 (128.17)**	**0.003**
*IL-10*	**56.560 (9.53)**	**18.219 (4.79)**	**0.011**
TNF-α	10.214 (6.50)	16.812 (8.12)	0.576
IFN-γ	120.787 (20.05)	137.933 (23.89)	0.602
*GM-CSF*	**27.060 (3.82)**	**66.130 (7.63)**	**0.004**
*Angiogenin*	**881.4542 (106.96)**	**1397.6704 (103.33)**	**0.013**
*ENA78*	**160.618 (28.74)**	**270.255 (27.36)**	**0.033**
*GRO-α*	**256.152 (27.78)**	**461.5086 (65.09)**	**0.027**
*MMP-3*	**75.897 (7.49)**	**128.3126 (15.34)**	**0.022**
*MMP-10*	**6.284 (3.15)**	**18.566 (2.58)**	**0.023**
*TIMP-2*	**4723.167 (190.92)**	**8402.9399 (505.84)**	**< 0.001**

SASP, senescence-associated secretory phenotype. Cytokines demonstrating significant differences in expression are given in boldface. Cytokine expression is measured as arbitrary units (AU). *N* = 2 biological and 2 technical replicates per sample. SE, standard error

### Changes in splicing factor expression and patterns of alternative splicing in senescent astrocytes

We have previously demonstrated changes in splicing factor expression in senescent primary human cells of different lineages (Holly et al., 2013, Latorre et al., 2017, Latorre et al., 2018b, Latorre et al., 2018c). Similar changes were also apparent in senescent astrocytes, where 10/20 of the splicing factors tested demonstrated lower expression in late passage cells compared with earlier passage cells (Table 3). Both *HNRNP* splicing inhibitors and serine-arginine (SR) rich splicing activator transcripts demonstrated dysregulation; 4/8 (50%) transcripts encoding splicing inhibitors, 3/8 splicing activator transcripts, and 3/4 (75%) of core spliceosomal transcripts demonstrated changed expression in senescent astrocytes. Most genes selected for study were expressed in astrocytes; only *AQP4M1*, *EAAT2A*, and *EAAT2B* were not. Eight of the remaining 13 transcripts demonstrated changes to their splicing patterns (Table 4).

**Table 3:** Comparison of splicing factor levels in senescent and non-senescent astrocytes

Splicing factor	Early passage astrocytes	Late passage astrocytes	*p* value
*AKAP17A*	1.035 (0.090)	1.01 (0.044)	0.077
***HNRNPA0***	**1.024 (0.077)**	**0.65 (0.031)**	**0.005**
*HNRNPA1*	1.236 (0.340)	1.863 (0.416)	0.449
*HNRNPA2B1*	1.343 (0.353)	0.6 (0.108)	0.257
***HNRNPD***	**1.094 (0.180)**	**0.646 (0.048)**	**0.03**
***HNRNPH3***	**1.071 (0.130)**	**0.743 (0.037)**	**0.003**
*HNRNPK*	1.153 (0.176)	1.37 (0.209)	0.551
***HNRNPM***	**1.021 (0.068)**	**0.66 (0.067)**	**0.012**
*HNRNPUL2*	1.074 (0.148)	0.784 (0.106)	0.089
***IMP3***	**1.088 (0.160)**	**0.62 (0.11)**	**0.035**
***LSM14A***	**1.042 (0.113)**	**0.315 (0.047)**	**0.005**
*LSM2*	1.377 (0.426)	0.369 (0.045)	0.065
***PNISR***	**1.041 (0.098)**	**0.777 (0.030)**	**0.005**
***SF3B1***	**1.018 (0.070)**	**0.716 (0.030)**	**0.018**
*SRSF1*	1.06 (0.140)	0.885 (0.038)	0.065
*SRSF2*	1.038 (0.095)	1.017 (0.045)	0.121
*SRSF3*	1.077 (0.175)	0.697 (0.032)	0.095
*SRSF6*	1.07 (0.126)	0.762 (0.104)	0.098
***TRA2B***	**1.022 (0.081)**	**0.757 (0.057)**	**0.031**
***SRSF7***	**1.119 (0.198)**	**0.51 (0.063)**	**0.015**

Values given refer to the mean expression of each splicing factor in either early or late passage cells. Values in parentheses are the standard error of the mean. Splicing factors demonstrating significant differences in expression are given in boldface. *N* = 3 biological and 3 technical replicates per sample

**Table 4:** Comparison of alternative isoforms of selected brain or senescence genes in senescent and non-senescent astrocytes

	Early passage astrocytes	Late passage astrocytes	*p* value
***GFAPΑ***	**0.809 (0.208)**	**0.062 (0.013)**	**0.0021**
*sKLOTHO*	1.155 (0.209)	0.498 (0.275)	0.131
***mKLOTHO***	**1.059 (0.122)**	**0.381 (0.012)**	**0.005**
*AQPM23*	1.252 (0.459)	1.067 (0.434)	0.784
***TAU3***	**1.044 (0.114)**	**0.603 (0.043)**	**0.023**
*TAU4*	1.23 (0.674)	1.542 (0.196)	0.679
***PSEN2***	**1.009 (0.12)**	**1.921 (0.158)**	**0.01**
***CDKN2A-p14***^***ARF***^	**1.043 (0.044)**	**0.532 (0.048)**	**0.001**
***CDKN2A-p16***^***Ink4A***^	**1.061 (0.076)**	**2.133 (0.127)**	**0.002**
***p21a***	**1.079 (0.085)**	**1.666 (0.087)**	**0.009**
***p21b***	**1.016 (0.095)**	**2.041 (0.077)**	**0.001**
*TP53*	0.974 (0.052)	1.038 (0.082)	0.541
*ATM*	0.926 (0.097)	0.612 (0.069)	0.057

Values given refer to the mean expression of each splicing factor in either early or late passage cells. Values in parentheses are the standard error of the mean. Transcripts demonstrating significant differences in expression are given in boldface. *N* = 3 biological and 3 technical replicates per sample

### Association of senescence-related transcripts with cognitive decline in a longitudinal human population

**Fig. 2:**
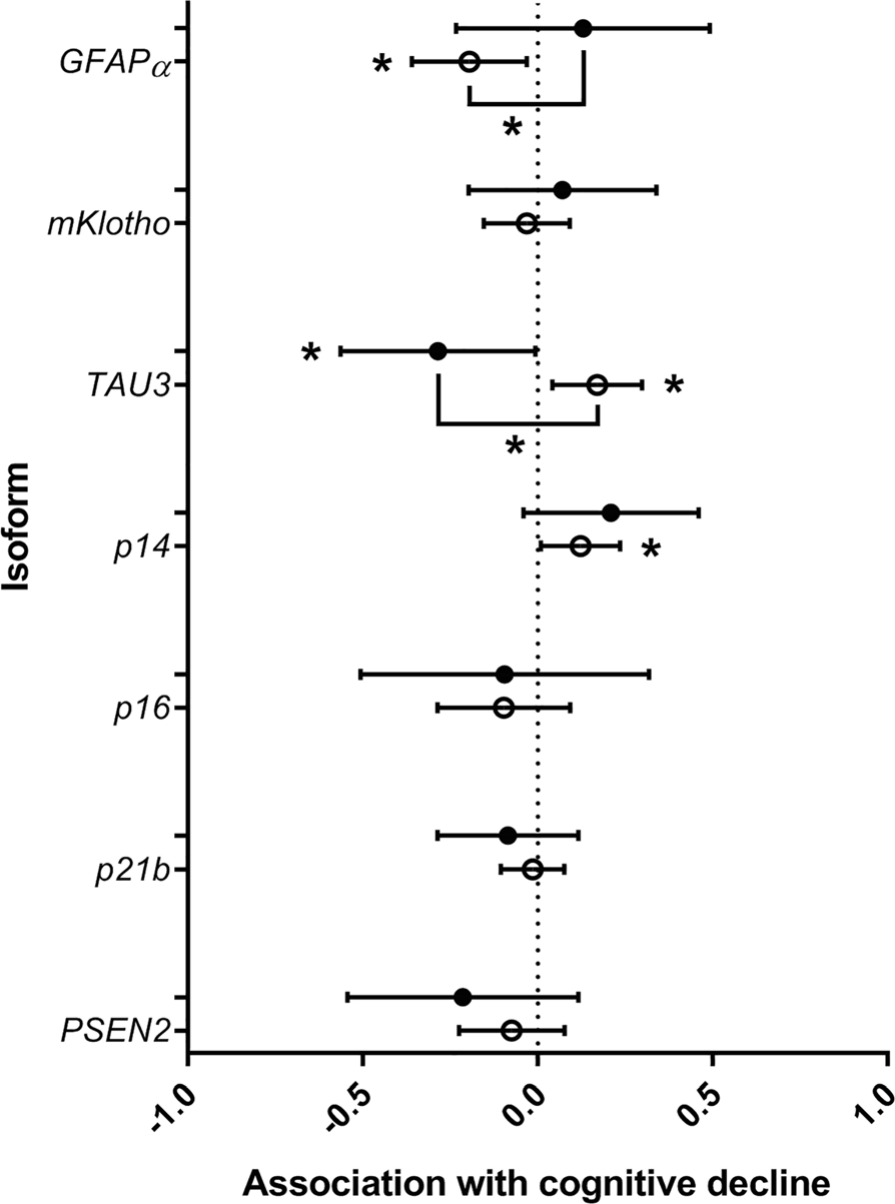
The association between isoform expression and cognitive decline in the InCHIANTI study of ageing. The graph indicates the associations between peripheral blood expression of alternatively expressed transcripts of genes with known links with neurodegenerative disease or cellular senescence and mild or severe cognitive decline is given in this figure. Mild decline is denoted by open circles; severe decline is denoted by closed circles. *p < 0.05. The null association point is given by the dotted line. Beta coefficients of association are given on the *X* axis and transcript identity is given on the *Y* axis. Data are from 197 participants classified as either mild cognitive decline (a reduction of 2–8 points in MMSE performance over a 3–7-year period) or severe decline (a reduction of 9–22 points in MMSE performance over the same period). Statistical differences in transcript expression between mild and severe decline are also given

We next assessed whether any of the transcripts demonstrating senescence-related changes in aged primary human astrocytes were associated with cognitive decline as assessed by change in MMSE score between FU3 and FU4, in peripheral blood mRNA from individuals in the InCHIANTI study of ageing. Of the 8 transcripts demonstrating associations with senescence in late passage astrocytes, *TAU3*, *GFAPα*, m*KLOTHO CDKN2A*(*p14*^*ARF*^), *CDKN2A*(*p16*^*INK4A*^), *CDKN1A*(*p21a*), *CDKN1A*(*p21b*), and *PSEN2* were also expressed in peripheral blood, and suitable for ongoing analysis. *CDKN2A* (*p14*^*ARF*^) and *TAU3* were positively associated with mild cognitive decline (*CDKN2A* (*p14*^*ARF*^) beta coefficient 0.122, 95% CI 0.01 to 0.24; *p* = 0.04, *TAU3* beta coefficient 0.170, 95% CI 0.042 to 0.297; *p* = 0.01), whereas *GFAPα* was negatively associated with mild cognitive decline (beta coefficient − 0.196 (95% CI − 0.36 to 0.032; *p* = 0.02; Fig. 2; Table 5). Interestingly, the only association we found with severe cognitive decline was a negative association with *TAU3* (beta coefficient − 0.286, 95% CI − 0.56 to 0.04; *p* = 0.04). *GFAPα* and *TAU3* demonstrated significant differences in level of isoform expression between mild and severe cognitive decline (Fig. 2, Table 5).

**Table 5:** Association between blood-based isoform production, induced by alternative splicing and cognitive decline in participants from the InCHIANTI population study of ageing. The table below gives the beta coefficients, 95% confidence intervals (95% CI), and *p* values for the association between candidate transcript expression and cognitive decline as assessed by change in MMSE for 197 individuals in a 3–7-year period. Mild cognitive decline is categorised as a decline of between 2 and 8 points in MMSE between FU3 and FU4, whereas severe cognitive decline is characterised as a decline of between 9 and 22 points. Transcripts demonstrating significant differences in expression are given in boldface

Mild cognitive decline (2–8 point decline in MMSE)				Severe cognitive decline (9–22 point decline in MMSE)				Difference between mild and severe decline in MMSE			
Isoform	Beta	95% CI	*p* value	Isoform	Beta	95% CI	*p* value	Isoform	Beta	95% CI	*p* value
***GFAPα***	**− 0.196**	**− 0.36 to 0.03**	**0.02**	GFAPα	0.129	− 0.23 to 0.49	0.48	**GFAPα**	**0.363**	**0.08 to 0.65**	**0.01**
*mKLOTHO*	− 0.032	− 0.16 to 0.09	0.61	mKLOTHO	0.070	− 0.20 to 0.34	0.61	mKLOTHO	0.093	− 0.18 to 0.37	0.50
***p14***	**0.122**	**0.01 to 0.24**	**0.04**	p14	0.209	− 0.04 to 0.46	0.10	p14	0.069	− 0.18 to 0.32	0.59
*P16*	− 0.097	− 0.29 to 0.09	0.31	P16	− 0.095	− 0.51 to 0.32	0.65	P16	− 0.029	− 0.45 to 0.40	0.89
*P21b*	− 0.015	− 0.11 to 0.08	0.74	P21b	− 0.085	− 0.29 to 0.12	0.40	P21b	− 0.030	− 0.22 to 0.16	0.75
*PSEN2*	− 0.075	− 0.23 to 0.08	0.33	PSEN2	− 0.215	− 0.55 to 0.12	0.20	PSEN2	− 0.133	− 0.47 to 0.20	0.43
***TAU3***	**0.170**	**0.04 to 0.30**	**0.01**	**TAU3**	**− 0.286**	**− 0.56 to − 0.01**	**0.04**	**TAU3**	**− 0.460**	**− 0.75 to − 0.17**	**0.01**

## Discussion

The accumulation of senescent cells as a result of repeated cell stresses is thought to be a major contributor to ageing and age-related disease. The ablation of senescent cells is able to bring about improvements in multiple age-related phenotypes in animal models (Baker and Petersen, 2018, Baker et al., 2011). Furthermore, correlations exist between circulating levels of senescence markers such as p16^INK4a^ and HMGB2 and functional status in humans (Lawrence et al., 2018). Suppression of senescent cell characteristics such as SASP using the flavonoid apigenin has also been demonstrated to reduce aggressive behaviour in breast cancer cells, providing further evidence reduction of senescent cell load may bring benefits for age-related diseases (Perrott et al., 2017).

Age-related dysregulation of alternative splicing may contribute to the accumulation of senescent cells during ageing. Alternative splicing, a key part of the molecular stress response that is a critical mediator of cellular plasticity and adaptability, has been shown to be disrupted during human ageing; restoration of splicing factor expression is able to reverse cellular senescence and bring about rejuvenation of multiple cell types in culture. We hypothesised that senescent astrocytes would display differential expression of splicing regulatory factors and altered patterns of alternative splicing in vitro, and that some of these isoform changes may be detectable in peripheral blood and show statistical associations with cognitive phenotypes in human populations. We demonstrated that 50% of splicing factor transcripts we measured demonstrated dysregulated expression in senescent astrocytes, and splicing changes were evident for almost half of the alternatively spliced genes in an a priori panel of candidate genes. Eight of the 13 candidate isoforms demonstrated splicing alterations in senescent cells, and were also expressed in human peripheral blood. Of these, 3 (*TAU3*, *GFAPα*, and *CDKN2A* (*p14*^*ARF*^)) were associated with mild cognitive decline in ageing humans. The *TAU3* isoform was also associated with severe cognitive decline, but in an opposing direction. Our data are consistent with a model, whereby age-related splicing factor changes may lead to splicing patterns for genes with roles in brain function or senescence, which may influence the development of cognitive decline in the human population. The links between splicing factor changes and isoform changes are impossible to predict from global measures. Each individual splice site in each gene is regulated by a unique and specific combination of activators and inhibitors which determine its usage or not, which makes prediction impossible from levels alone. What our data do indicate is that senescent astrocytes have disrupted expression of many splicing factors, which would be predicted to alter the splicing patterns. We have demonstrated that this holds true for a number of genes important in senescence or in astrocyte function.

Some of the adverse effects of the presence of senescent cells arise because of the paracrine signalling effects of the SASP on neighbouring cells. The presence of senescent cells has been suggested to contribute to shortened overall lifespan (Baker et al., 2016), and clearance of such cells was able to bring about a delay in the appearance of ageing phenotypes in ageing mice (Baker et al., 2011). Other groups have reported increased lifespan, rejuvenation of ageing phenotypes such as thinning fur and improved kidney function in old mice that have undergone targeted removal of senescent cells (Baar et al., 2017). Ablation of senescent glial cells has been demonstrated to lead to a reduction of Tau-dependent pathology and improve cognitive function in mice (Bussian et al., 2018). Accordingly, we observe the generation of a strong SASP in astrocytes that have undergone replicative senescence, which may be contributory to the inflammatory increases evident in the pathophysiology of neurodegeneration. The SASP profile exhibited by the primary human astrocytes does not match that profile seen in other cell lines from our previous work (Latorre et al., 2017, Latorre et al., 2018b, Latorre et al., 2018c); however, cell type specificity in SASP has previously been described (Coppe et al., 2010). The reasons behind these variations are unclear, but may reflect histologically discrete programmes, dysregulation patterns specific to existing transcriptomic profiles, or simply arbitrary patterns of cytokine production which represent the stochastic molecular dysregulation which is occurring. Our observations, together with the marked changes in splicing factor expression, are suggestive that the intersection between disrupted regulation of splicing, cellular senescence, and its associated inflammatory phenotype in astrocytes may contribute to astrocyte dysfunction and have some bearing on eventual cognitive decline. Similar changes may also be occurring in other important proliferative brain cell types such as microglia.

The changes in astrocyte splicing factor expression we have identified here probably do not act directly on splicing regulation in blood, but rather reflect related cognition-associated changes to splicing factor expression in blood as we have recently described (Lee et al., 2019). Similarly, the altered expression of alternatively expressed transcripts in blood may be reflective of similar changes that we demonstrate here also occur in astrocytes. We identified positive associations between *CDKN2A* (*p14*^*ARF*^) and *TAU3* expression in peripheral blood and mild cognitive decline, and a negative correlation between *GFAPα* expression and mild cognitive decline. The associations between GFAPΑ and *CDKN2A* (*p14*^*ARF*^) were not apparent in severe cognitive decline, although this may reflect low power imposed by the inherent variability of human biological samples, since p14 expression in severe decline is trending in the same direction. The association of *TAU3* expression with severe cognitive decline was still evident, but was negatively, rather than positively correlated. GFAPα expression also demonstrates opposing direction of effect between mild and severe decline. This may represent differences in cell subtype populations, or altered cell characteristics between disease states. GFAPα, which along with other intermediate filaments forms the cytoskeleton, is important for signal transduction and structural properties (Thomsen et al., 2013). *GFAPα* is highly expressed during key developmental stages during gestation and has also been observed to have elevated expression levels in brain damage and a range of neurological diseases (Thomsen et al., 2013). Some studies have reported no change in whole blood GFAPα protein levels and hypothesised that without rapid astroglial destruction, GFAPα levels may not climb to a detectable level and thus may not be a good indicator of neurological pathology (Mayer et al., 2013). Such studies however may not detect changes at the level of isoforms, if appropriate antibodies capable of specifically identifying individual splice variants are not used.

Increased levels of *CDKN2A* (*p14*^*ARF*^) may be reflective of an increased load of senescent cells, and is one of two proteins produced by alternate reading frame of the *CDKN2A* locus (Aram et al., 2016). p14^ARF^ inhibits the activity of MDM2, a protein which sequesters the p53 protein (Wei et al., 2001). Once p53 stabilises and accumulates, it can trigger DNA repair or the apoptosis program of cell death (Fridman and Lowe, 2003). The consequences of increased p14^ARF^ levels are complex and in places conflicted. It has been demonstrated to induce either cellular senescence or apoptosis in a *p53*-dependant manner (Aram et al., 2016). There have also been some reports that although *CDKN2A* (*p14*^*ARF*^) transcripts are upregulated in senescent cells, *TP53* and *MDM2* levels can remain unchanged (Wei et al., 2001). Ectopic expression of p14^ARF^ is capable of inducing senescence but overexpression must be maintained to commit cells to a senescent state (Tokarsky-Amiel et al., 2013). *TAU3* transcript expression in the blood was observed to be correlated positively with mild cognitive decline, but interestingly, negatively correlated with severe cognitive decline. Microtubule-associated protein Tau (*MAPT*) is an alternatively spliced regulator of microtubule dynamics—which is essential for cellular functions from structure to transport (Morris et al., 2011). An ever-growing body of research in human and animal models of tauopathies is demonstrating that the delicately balanced 2:1 3R/4R TAU ratio (Chen et al., 2010), which is achieved through alternative splicing of exon 10, is imperative for maintaining healthy function in cells in the brain, and shifts to disrupt this balance in either direction can be catastrophic (Schoch et al., 2016, Espindola et al., 2018, Avale et al., 2013, Chen et al., 2010). A number of neurodegenerative diseases, including Alzheimer’s and frontotemporal dementia (FTD), have had tau isoform balance (specifically 3R and 4R isoforms) implicated in their pathophysiology (Jakes et al., 1991, Lacovich et al., 2017, Morris et al., 2011). These include those examples with premature cognitive decline (Hu et al., 2007). The precise mechanisms by which TAU isoforms specifically contribute to each neurological pathology are still being investigated. The directionality of association between *TAU3* transcript levels and mild and severe decline is consistent with existing literature suggesting imbalances in 3R/4R isoform ratios, rather than absolute isoform levels per se, are identified in cases of dementia and examples of neurodegenerative disease (Chen et al., 2010, Iqbal et al., 2010).

Our finding of dysregulated splicing factor expression in human primary astrocytes is novel and consistent with reports from senescent cells of other tissue types (Holly et al., 2013, Latorre et al., 2017, Latorre et al., 2018b, Latorre et al., 2018c). Our study benefits from a systematic cells-to-populations approach including primary human cell lines and an exquisitely characterised longitudinal population study. Weaknesses of this study are the initial assessment in isolated astrocytes, which may not represent the holistic nature of the cytological and molecular mechanisms involved in cognitive decline and may not capture the extent of cross talk between other cell types in the human brain. It also need to be recognised that the astrocytes used in this study derive from a single donor, and that differences reported here would benefit from confirmation in individuals of different genetic background. Cell lines from a wider variety of donors may return more genes of interest; however, these tissues are rare and difficult to acquire. In addition, our priori list of genes does not include many other isoforms and genes involved in the process. Several of the isoforms selected for study are also not strictly specific for astrocytes. This is particularly true of the senescence genes tested, which reflect players in a more global mechanism. Our scope was narrowed by the need for these genes to be expressed in the blood, for assessment of association with living population. Further work may benefit from the whole transcriptome sequencing and transcriptional profiling in a number of different cell types.

Our data are consistent with a model by which accumulation of senescent astrocytes (and doubtless other important brain cell subtypes), their associated disrupted splicing patterns, and the increased inflammatory microenvironment may contribute to premature cognitive decline. Inflammation and related cellular stresses are capable of activating cell signalling pathways and lead to further dysregulation of splicing factor expression (Latorre et al., 2018a), so it is possible that an auto-regulated feedback loop involving SASP-derived increases in inflammation, dysregulated splicing regulation, and subsequent further increases in senescent cell load may occur as a result of positive feedback. The idea that some of the features of cognitive decline could therefore arise from dysregulated splicing of genes important in the support cells of the brain requires further exploration. This could be explored further by the selective manipulation of specific isoform levels, followed by assessment of effects on astrocyte cell kinetics or astrocyte function in cells and in systems. Our observations suggest that the role of splicing factor expression and dysregulated alternative splicing in cognitive decline may represent an interesting line of future investigation.
